# Insights Into Development and Progression of Idiopathic Pulmonary Fibrosis From Single Cell RNA Studies

**DOI:** 10.3389/fmed.2020.611728

**Published:** 2020-12-16

**Authors:** Julia Nemeth, Annika Schundner, Manfred Frick

**Affiliations:** Institute of General Physiology, University of Ulm, Ulm, Germany

**Keywords:** fibroblast, alveolar, lung, IPF, ATII cells, scRNA sequencing

## Abstract

Idiopathic pulmonary fibrosis (IPF) is a progressive and fatal lung disease with limited therapeutic options. The current model suggests that chronic or repetitive “micro-injuries” of the alveolar epithelium lead to activation and proliferation of fibroblasts and excessive extracellular matrix (ECM) deposition. Disruption of alveolar type II (ATII) epithelial cell homeostasis and the characteristics of mesenchymal cell populations in IPF have received particular attention in recent years. Emerging data from single cell RNA sequencing (scRNAseq) analysis shed novel light on alterations in ATII cell progenitor dysfunction and the diversity of mesenchymal cells within the fibrotic lung. Within this minireview, we summarize the data from most recent human scRNAseq studies. We aim to collate the current knowledge on cellular plasticity and heterogeneity in the development and progression of IPF, effects of drug treatment on transcriptional changes. Finally, we provide a brief outlook on future challenges and promises for large scale sequencing studies in the development of novel therapeutics for IPF.

## Introduction

Idiopathic pulmonary fibrosis (IPF) is a progressive, irreversible, and usually fatal respiratory disease with limited therapeutic options (1). The current model suggests that IPF is a result of repeated epithelial cell injury and inadequate repair of the alveolar epithelium that leads to excessive fibroblast activity and lung fibrosis (2). Alveolar type 2 epithelial (ATII) cells function as progenitor cells that maintain homeostasis of the epithelium and repair the damaged epithelium after injury (2–6). Disruption of ATII cell homeostasis and loss of facultative progenitor capacity are drivers for lung fibrosis (1, 7, 8). The aberrantly activated lung epithelial cells secrete mediators that lead to fibroblast proliferation and differentiation to highly active myofibroblasts, which deposit excessive amounts of extracellular matrix (ECM) (9). This results in overall remodeling of the alveolar structure, formation of scar tissue, thickening of the alveolar septae and an increase in tissue stiffness (10, 11). The inability to resolve fibrotic processes results in ongoing activation of fibroblasts creating a vicious cycle with fatal outcome for the patient.

Disruption of ATII cell homeostasis and the characteristics of mesenchymal cell populations in IPF have therefore received particular attention in recent years. Apart from ATII cells and alveolar fibroblasts, recent studies also suggested that other cell types may have an important role in IPF initiation. This entails further, recently identified, sub-populations of specific epithelial and/or mesenchymal cells and possible contributions of other cells in the distal lung, most notably macrophages (12–15).

Overall, detailed cellular contributions to the onset and progression of IPF are still not fully elucidated. The advent of scRNA technology has increased our understanding of lung cell heterogeneity and cellular interplay in healthy and diseased lung tissue. Initial efforts were mainly conducted in mice, which also offer cell-type specific lineage tracing to study disease onset and progression (6, 16–18). However, the human lung contains many structural and cellular differences to the mouse lung and, although the alveolus is one of the most conserved regions between mouse and human lungs, it is still debated whether all cells in the human distal lung have corresponding counterparts in the murine lung (5, 8, 19). Mice appear to lack a substantial proportion of the molecularly defined human lung cell populations, including sub-populations of alveolar epithelial and mesenchymal cells (20). Hence, mice might not accurately recapitulate human disease if critical cell types are not present. In particular, mouse models of IPF, including the widely used bleomycin model, still do not recapitulate all features of IPF pathogenesis (21). It is therefore indispensable to extend our knowledge of the human lung. Recent efforts, have led to ever increasing datasets aiming at establishing a detailed cellular atlas of the human lung. These novel datasets are a valuable source to identify and map disease origins, progression and potential therapeutic targets benefiting patients (20, 22). Recently, scRNAseq approaches were also applied in studying transcriptional changes in IPF and extended our knowledge of the human IPF transcriptome (Table 1). Within this review, we summarize the data from most recent scRNAseq studies aiming to collate the current knowledge on cellular plasticity and heterogeneity in the development and progression of IPF in humans as well as effects of drug treatment on transcriptional changes. We also provide a brief outlook on future challenges and promises for large scale sequencing studies in the development of novel therapeutics for IPF.

**Table 1:** Overview of the main scRNAseq studies included in this review.

**References**	**Analyzed cell type(s)**	**Cell separation technology**	**Sequencing** **platform**	**Number of donors**	**Number of analyzed cells**	**Cell clusters identified**
Xu et al. (23)	Epithelial cells	FACS (CD326, CD31, CD45, HTII-280)	SMART Seq	Healthy 3 IPF 6	215 healthy 325 IPF	4 epithelial
Xi et al. (24)	ATII	FACS (CD326, CD31, CD45, HTII-280)	Not specified	Not specified	23–72	5 alveolar epithelial
Reyfman et al. (12)	Macrophages Alveol. epithelium	FACS	Not specified	Healthy 8 IPF 4	76,070	22 total inlud. 4 macrophages 5 alveolar epithelial
Adams et al. (13)	Fibroblasts Epithelial cells Endothelial cells Immune cells	No additional separation	Chromium Seq	Healthy 29 IPF 32	312,928	38 total inlud. 7 fibroblast 9 epithelial 5 endothelial 14 immune
Morse et al. (25)	Macrophages	Not specified	Chromium Seq	Healthy 3 IPF 3	17,231 healthy 30,540 IPF	23 total inlud. 3 macrophages
Vieira Braga et al. (22)	Epithelial cells Immune cells	No additional eparation	Chromium Seq	Healthy 9 Asthma 6	36,931	21 total inlud. 10 epithelial 6 main immune
Valenzi et al. (26)	Fibroblasts	No additional separation	Not specified	Healthy 4 SSc-ILD 4	56,196	4 fibroblast
Liu et al. (15)	Fibroblasts	No additional separation	Chromium Seq	Not specified	Not specified	8 fibroblast
Travaglini et al. (20)	Epithelial cells	FACS/MACS (CD326, CD31, CD45)	Chromium Seq	Not specified	~75,000	58 total cells inlud. 15 epithelial 9 endothelial 9 stromal 25 immune
Mayr et al. (27)	Fibroblasts	No additional separation	Drop Seq	Healthy 11 IPF 3	41,888	45 total
Carraro et al. (28)	Epithelial cells	FACS (CD326, CD235a, CD45, CD31, Cd271, CD66)	Chromium Seq	Healthy 6 IPF 7	Not specified	37 total inlud. 4 basal 2 ciliated 5 secretory
Habermann et al. (14)	Fibroblasts Epithelial cells	MACS (CD235a, CD45)	Chromium Seq	Healthy 10 PF 20	114,396	31 total inlud. 12 epithelial 4 fibroblasts
Tsukui et al. (29)	Fibroblast	FACS (CD31, CD45, CD235a, CD326)	Chromium Seq	Healthy 3 IPF 3	48,587	7 fibroblast

## Diversity of Lung Cell Types and Regulatory Mechanisms During Health and Disease

### Epithelial Cells

Inadequate alveolar epithelial repair is believed to constitute the initial trigger for fibrotic remodeling in IPF. Within the alveolar epithelium, constituted of alveolar type I epithelial (ATI) and ATII cells, ATII cells are considered self-renewing stem cells that also regenerate ATI cells after injury (3). The contribution of ATI cells to alveolar repair is thought to be very limited (30).

**Figure 1:**
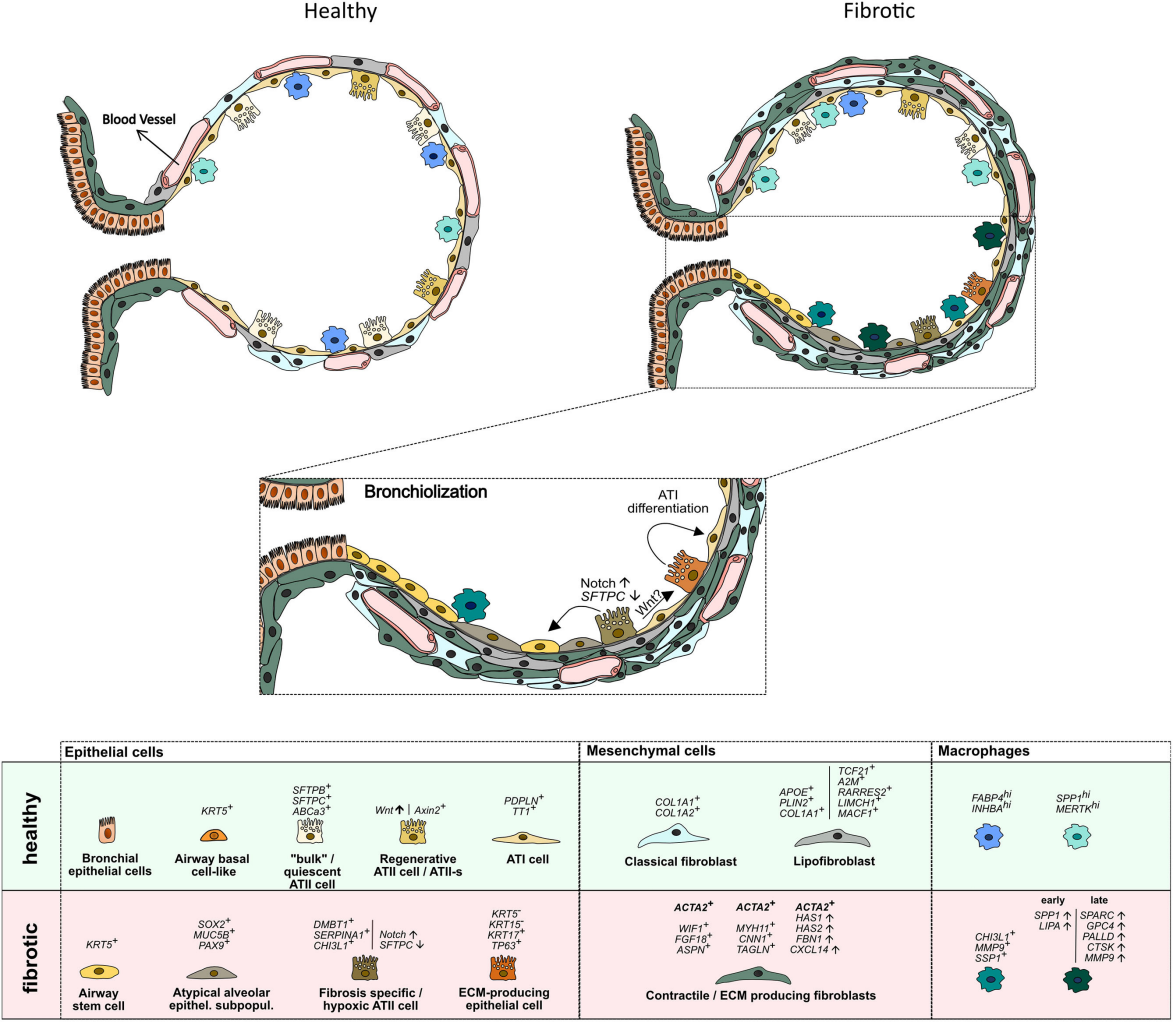
Schematic representation of cell types identified by scRNA sequencing in the distal lung of healthy and IPF donors. Vertical lines separate marker genes identified by different authors for similar cell types. Arrows depict presumed transdifferentiation pathways and respective signaling pathways. For details see main text.

Recent scRNAseq studies identified sub-lineages within the ATII cell population in mice and humans that serve as facultative progenitor cell population to regenerate alveoli after injury. In contrast to “bulk”, quiescent ATII cells, these ATII signaling (ATII-s) cells are Wnt-active (20), and in mice, were initially characterized by expression of the transcriptional target of Wnt signaling, *Axin2* (6, 18). A recent scRNAseq study directly comparing lung tissue obtained from (healthy) transplant donors and recipients with pulmonary fibrosis, however, was unable to identify this sub-population in humans, albeit finding *AXIN2* expression in ATII cells. Interestingly, this study reported non-overlapping expression of Wnt ligands and *AXIN2* in epithelial cells, suggesting that Wnt secretion and response were separated to specific cells. These authors also found small clusters of ATII cells that were distinct between healthy and fibrotic donors, with fibrosis-specific ATII cells expressing *DMBT1, SERPINA1*, and *CHI3L1*, genes previously associated with pulmonary fibrosis (12). The study by Reyfman et al. also identified rare cell populations including airway stem cells and senescent cells to emerge during fibrosis (12). Similarly, principal component analysis (PCA) and whole-genome unsupervised hierarchical clustering of scRNAseq-derived transcriptomes clearly separated ATII cells in a study comparing normal and fibrotic lungs. ATII cells from fibrotic lungs revealed a hypoxic sub-population with activated Notch, suppressed surfactant protein C (*SFTPC*), and transdifferentiation towards a *KRT5*^+^ airway basal cell-like state, whereas Wnt activity prior to Krt5 activation favors differentiation toward ATII cells, leading to normal alveolar epithelial repair (24). Atypical sub-populations of alveolar epithelial cells expressing airway-associated genes (*SOX2, MUC5B*, and *PAX9*), were also reported in explant tissue of patients undergoing transplant for end-stage IPF (23). Identification of airway stem cells within fibrotic lesions therefore matches the histological observation that epithelial cells with airway markers accumulate in fibrotic regions of human lungs, in a process termed bronchiolization (Figure 1) (31). Epithelial cells with airway markers are also found in micro-honeycomb cysts, another feature of fibrotic remodeling in human lung (32).

Basal cells are the major stem cells in the human airways that self-renew and can give rise to all other cell types of the airways (33). scRNAseq studies have identified various clusters of basal cells in humans, that might differ in their ability to function as stem cells (20, 22, 28). Carraro et al. reported a sub-population of *CD66*^+^ secretory cell primed basal (SPB) cells, that share a molecular signature with basal cells recovered from fibrotic lung tissue (28). SPB cells from IPF were enriched in *CEACAM6* expression and could be distinguished from normal SPB cells by their gene signature. IPF SPB cells expressed genes previously identified as serum biomarkers for higher risk of mortality and proposed to be involved in disease development (*MUC1, MMP7, ICAM1*). Two scRNAseq studies published this year, further added to the notion of aberrant basaloid cells, expansion of airway epithelial cells and reduction in alveolar epithelial cells in IPF lungs (13, 14). Habermann et al. observed a remarkable shift in epithelial cells by comparing IPF with normal lung tissue. Cells expanded in IPF lungs were airway epithelial cells (*KRT5*^+^ basal cells, *FOXJ1*^+^ ciliated cells, and secretory cells), whereas alveolar epithelial cells were recovered to a lower extend (14). They also identified a novel *KRT5*^−^*/KRT15*^−^*/KRT17*^+^*/TP63*^+^, pathologic ECM-producing epithelial cell population which was drastically increased in IPF lungs. This new cell population was in parallel described by Adams et al. (13). Both studies linked the new cell type with the co-expression of basal epithelial, mesenchymal, and senescence markers as well as developmental transcription factors. The origin of the *KRT5*^−^*/KRT17*^+^ was either traced to ATII or Club cells (14) or myofibroblast foci (13). Overall, this leads to the hypothesis that ATII as well as Club cells can differentiate into an intermediate *KRT5*^−^*/KRT17*^+^ epithelial cell subtype which can further differentiate into ATI cells, which is in accordance to other studies describing an intermediate alveolar epithelial cell population (34, 35). These reports are in line with the hypothesis that an abnormal differentiation program is initiated in the tissue microenvironment of IPF in which the proximal-peripheral patterns of cell differentiation are disrupted. This results in many respiratory epithelial cells acquiring aberrant, multilineage-like states and some individual cells sharing the characteristics of both conducting airway and alveolar epithelial cells (23). The mobilization/activation of airway stem cells may be a potential mechanism to maintain tissue integrity. However, their differentiation into airway rather than alveolar phenotypes potentially accounts for the dysplastic structures observed in fibrotic lung tissues (36). These structural defects are considered to be secondary to epithelial progenitor cell dysfunction and defective epithelial-mesenchymal signaling (28).

### Mesenchymal Cells

Mesenchymal cells in the alveolus serve multiple functions to maintain alveolar integrity. They generate and remodel the ECM, co-regulate the vasculature, help maintain and restore the epithelium, act as sentries for the immune system, and have been found to have trophic interactions with alveolar epithelial cells (37). After epithelial injury, fibroblasts become activated and induce many developmental pathways that are important for proliferation, ECM deposition, and chemokine and cytokine secretion to mediate tissue repair (8, 38). Aberrant proliferation and excessive ECM deposition result in fibrotic tissue remodeling and increased tissue stiffness found in lungs from IPF patients.

Historically, mesenchymal cells were mainly divided into fibroblasts, smooth muscle cells (SMCs) and pericytes, a derivative of fibroblasts with myofibroblast characteristics, that is closely associated with capillaries (39). Recent studies, including scRNAseq efforts, have vastly increased the heterogeneity of mesenchymal cells found in the human lung (12–15, 20, 22, 26, 27).

A recent comprehensive droplet- and plate-based scRNAseq study identified 4 fibroblast clusters within alveolar tissue samples. One cluster expressing classical fibroblast markers (*COL1A1, COL1A2*), two clusters enriched for *ACTA2*, a canonical marker of myofibroblasts, and a fourth cluster representing lipofibroblasts (*APOE*^+^*, PLIN2*^+^*, COL1A1*^+^), a cell that's presence in the human lung has long been controversial (40). Myofibroblasts are of particular interest in the development and progression of IPF. Myofibroblasts help forming alveoli during development and restore tissue integrity after injury. However, they are also considered the primary drivers of ECM deposition in fibrosis, and the key effector cells in IPF combining of the synthesizing features of fibroblasts with the cytoskeletal contractile characteristics of SMCs (2, 41). Interestingly, the *ACTA2*^+^ “myofibroblast” population could be separated into two clusters. One *WIF1*^+^*, FGF18*^+^*, ASPN*^+^ cluster representing “classical” myofibroblasts localized to alveolar ducts. The other sub-population, however, showed higher expression of contractile genes (*MYH11, CNN1, TAGLN*) and was termed “fibromyocyte”. Both expressed a rich set of genes associated with TGF-β signaling (*LTBP1, LTBP2, ASPN, DPT, TGFBR3, TGFBI, SCX, MDFI*) (20).

In line, a study by Liu et al. also identified up to eight mesenchymal cell populations in lung tissue from healthy and IPF donors. This included intermediate fibroblasts, again two sub-populations of *ACTA2*^+^ myofibroblasts and a population of lipofibroblasts (*TCF21*^+^*, A2M*^+^*, RARRES2*^+^*, LIMCH1*^+^*, MACF1*^+^), and an *EBF1*^+^ sub-population. Concordantly, myofibroblasts expressed contractile genes (*MYH11*). This was increased in IPF. These authors also found that all mesenchymal subtypes contributed to the excessive ECM production in fibrosis and that there is little evidence for trans-differentiation of fibroblast subtypes and that fibroblast fate is determined early in lung development (15). This is supported by another recent study that suggested a continuous trajectory toward IPF archetypes co-occurring within fibroblasts and myofibroblasts, but not necessarily across them. This study identified two stromal populations in IPF and control lungs. Fibroblasts, characterized by expression of *CD34, FBN1, FBLN2, VIT*, and myofibroblasts consistently expressing *MYLK, NEBL, MYO10, MYO1D, RYR2*, and *ITGA8*. The IPF lung in addition contained a myofibroblast phenotype enriched with fibrillar collagens, and *ACTA2* and a fibroblast phenotype that exhibited increased expression of *HAS1, HAS2, FBN1*, and *CXCL14*. The authors suggest that pathological, *ACTA2*-expressing IPF myofibroblasts are not a discrete cell type, but rather one extreme pole of a continuum connected to a quiescent *ACTA*2-negative stromal population represented in control lungs (13). A *HAS1*^hi^ fibroblast population was also found in a study released at the same time and suggested to represent an invasive phenotype as it appeared restricted to the immediate subpleural regions (14) where fibrotic foci are usually found first.

Overall, these studies suggest that the heterogeneity of the fibroblast population increases in IPF lungs compared to controls, that transdifferentiation of fibroblast subtypes likely is not relevant in IPF and that fibroblasts are transcriptionally distinct from myofibroblasts. Maybe even all sub-populations may contribute to the excessive ECM production seen in fibrosis.

### Other Cells

Alveolar cell injury leads to inflammation and activation of immune cells. Murine models of bleomycin-induced fibrosis suggested that (monocyte-derived) macrophages could contribute to progression of fibrosis through overreactive responses to alveolar injury (42, 43). Recent scRNAseq reports also provide evidence for the involvement of macrophages in human fibrosis. A first study defined four macrophage clusters in IPF lungs in total. Two clusters including alveolar macrophages were equally represented in normal and fibrotic lungs. The other two clusters were almost exclusively found in lungs of patients with fibrosis. In combination with immunocytochemistry, *CHI3LI, MMP9*, and *SSP1* were suggested as markers for macrophages detected in IPF samples (12). A subsequent study found three discrete macrophage sub-populations, which were all present in normal and fibrotic lungs, one expressing monocyte markers, one highly expressing *FABP4* and *INHBA* (*FABP4*^hi^), and one highly expressing *SPP1* and *MERTK* (*SPP1*^hi^). Proliferation of *SPP1*^hi^ macrophages was strikingly increased in IPF lungs. Colocalization and causal modeling supported the role for these highly proliferative *SPP1*^hi^ macrophages in activation of IPF myofibroblasts in lung fibrosis (25). In line, a recent study also found a profibrotic macrophage archetype that gradually and sequentially shifted toward its most extreme terminus with IPF progression. Expression of *SPP1* and *LIPA* steadily increased relatively early along the trajectory with ECM remodeling genes *SPARC, GPC4, PALLD, CTSK*, and *MMP9* ramping up at later stages (13).

In addition, this recent study also identified changes in vascular endothelial cells in IPF lungs. A vascular endothelial cell population that expresses *COL15A1* was found in the IPF lung. This is transcriptomically indistinguishable from systemically supplied bronchial vascular endothelial cells detected in control lungs. However, in IPF lungs, these cells can readily be found in affected regions in the distal IPF parenchyma whereas these cells are restricted to the peri-bronchial vasculature in the normal lung and are never seen in the lung parenchyma. The authors speculate that the ectopic presence of these cells provides the cellular molecular correlate to an old notion that the bronchial vascular network is expanded throughout the IPF lung (13).

## NEW Insights From scRNAseq Studies Into Disease Progression and Biomarker Identification

scRNAseq studies have vastly increased our understanding of cell types resident in the distal lung and also highlighted differences in cell populations/states between healthy and IPF tissue (12–15, 20, 25, 28, 44, 45). Recent efforts aimed at extending these insights to a better understanding of mechanisms for the local and temporal disease progression or leveraging scRNAseq analysis to progress disease biomarker identification (27, 46). Subclassifying IPF tissue samples into three pathological defined groups of no fibrosis detectable, mild fibrosis and advanced fibrosis a recent study detected large changes in IPF patients' samples even before histological changes in the respective regions were detectable. Thirteen different gene expression tracks were identified and assigned to changes in mRNA and microRNA (regulators of transcription) expression within different cell types over the respective disease progression states (46). Combining scRNAseq of lung tissue with proteomic analysis of bronchoalveolar lavage (BAL) and plasma from healthy and fibrotic donors another study discovered that fluid proteome signatures were predictive of specific cell state changes in the lung. Markers of several pro-fibrogenic cell types were strongly increased in protein measurements of patients that were diagnosed with different forms of interstitial lung diseases (ILD) including patients with IPF when compared to non-ILD controls. These protein signatures correlated with diagnosis, lung function, smoking and injury status (27). scRNAseq data also offer the opportunity to identify transcription factors, signaling interactions and hormone/immune targets linked to disease onset and progression (20). Similarly, it offers the opportunity to identify novel diseases-related genes.

## scRNAseq to Understand Effects of Drug Treatment for IPF

To date, Nintedanib and Pirfenidone are the only approved drugs for treatment of IPF (47, 48). Two recent studies employed scRNAseq to investigate the effects of drug treatment on cells from IPF patients. A comparative transcriptomic approach on lung homogenates and lung fibroblasts derived from IPF patients treated with or without pirfenidone revealed that pirfenidone exerts the beneficial effects via its action on multiple pathways in both fibroblasts and other pulmonary cells. In fibroblasts, pirfenidone therapy predominantly affected growth and cell division pathways indicating a major cellular metabolic shift. *CEMIP*, linked to cell migration and ECM production, was downregulated in fibroblasts and lung homogenates (49). Nintedanib treatment resulted in 157 up- and 151 downregulated genes in IPF fibroblasts. Bioinformatics analysis revealed strong protein–protein interactions within these dysregulated genes, mostly involved in the pathways of cell cycle and mitotic cell cycle. Moreover, changes in expression of several miRNAs were identified, in particular upregulation of hsa-miR-486-3p which might repress expression of *DDX11, E2F1*, and *PLXNA4*, genes linked to fibroblast proliferation and reduction of angiogenesis (50). Further studies will be required to foster our understanding of drug treatment on individual cell populations in IPF. Corticosteroids are often used to dampen the effects of acute exacerbations in IPF, however, the benefit is still debated (51) and might even be contra-indicated in combination with immunosuppressive treatment (52). scRNAseq could potentially help in identifying beneficial or adverse effects of corticosteroid treatment on individual cell populations and delineate cellular target effects.

## Challenges and Future Perspectives

scRNAseq methods enable unbiased, high-throughput, and high-resolution transcriptomic analysis of individual cells. This provides an additional dimension to transcriptomic information relative to traditional methods that profile bulk populations of cells and are uniquely suited to look for heterogeneity within cell populations that might emerge during disease (53). However, scRNAseq approaches still have limitations and require cautions interpretation of data. In comparison with bulk RNAseq, the obtained data are noisier, complicating computational analysis and leading to a drop-out of weakly expressed genes (54). Likewise, identifying clusters based on delineating marker genes reported in earlier studies introduce a bias during the process of cell clustering (15). Although new analytical tools have been designed, methods resolving technical noise, considering expression variability and offering unbiased clustering of cells are still not available (55). In addition, cell isolation procedures, including up- and downstream processing, are highly variable between studies, limiting reproducibility and impacting on accuracy of the reported data sets often leading to over- or underrepresentation of specific cellular sub-populations. It will be a major challenge for the future to converge the various datasets and identify unifying marker sets to identify and specify individual cell types. In addition, the abundance of data generated in scRNAseq studies are currently lacking realistic methods of validation on the protein level due to the lack of methods for proteomics at this scale. Albeit, first attempts are under way link scRNAseq data and proteomic analysis to identify disease specific changes on the cellular level and their corresponding reflection in body fluid proteomes (27). Animal studies, despite their limitations, will also benefit in validating data from scRNAseq studies in experimental models. Some studies are already available that confirm the predicted alterations in cellular composition between healthy and fibrotic lung tissue. For example, the appearance of cells expressing airway stem cell markers in the alveolar region was associated with fibrosis in a murine bleomycin model (56).

Nevertheless, new tools enabling data analysis, visualization, and interpretation are being developed at a rapid pace and are readily available as “open source” tools (8). Within the last years, further development and optimization of new scRNAseq protocols are now enabling high throughput measurements (57–59). Novel technologies including RNA velocity and “spatial transcriptomics” can extend and complement scRNAseq studies and deepen our understanding of dynamic processes and/or spatial differences in lung development, cellular organization, and disease progression. RNA velocity, the time derivative of the gene expression state, is a high-dimensional vector that predicts the future state of individual cells (on a timescale of hours) and aids the analysis of developmental lineages and cellular dynamics (60, 61) while “spatial transcriptomics,” allows visualization and quantitative analysis of the transcriptome with high spatial resolution in individual tissue sections (62, 63). Such technologies can be particularly useful in investigating the onset and progression of IPF, a disease with great local heterogeneity (“fibrotic foci”) and rapid progression. Therefore, recent studies investigating lung cell identity and/or changes in fibrotic tissue have already started to apply such approaches (14, 29, 44, 56, 64, 65).

The rising number of sequenced cells and the number of patients per study are strengthening accuracy and resolution (Table 1). The reduction of costs and increase in throughput also facilitates scRNAseq studies to be performed at multiple time points, supporting the delineation of disease progression. Most datasets are publicly available and offer the opportunity to analyze combined datasets in the quest for discovering new therapeutic targets. In addition, exploiting these advancements in scRNAseq and combining them with complementary technologies including bulk RNASeq, imaging, and functional studies will further strengthen mechanistic insights into the onset and progression of IPF and aid in the development of new therapeutics.

## Author Contributions

JN, AS, and MF wrote the manuscript. All authors agree to be accountable for the content of the work.

## Conflict of Interest

The authors declare that the research was conducted in the absence of any commercial or financial relationships that could be construed as a potential conflict of interest.
